# Paricalcitol Inhibits Aldosterone-Induced Proinflammatory Factors by Modulating Epidermal Growth Factor Receptor Pathway in Cultured Tubular Epithelial Cells

**DOI:** 10.1155/2015/783538

**Published:** 2015-05-06

**Authors:** Jose L. Morgado-Pascual, Sandra Rayego-Mateos, Jose M. Valdivielso, Alberto Ortiz, Jesus Egido, Marta Ruiz-Ortega

**Affiliations:** ^1^Cellular Biology in Renal Diseases Laboratory, IIS-Fundación Jimenez Diaz, Universidad Autónoma Madrid (UAM), 28040 Madrid, Spain; ^2^REDINREN, Madrid, Spain; ^3^Department of Experimental Nephrology, Universitat de Lleida/Institut de Recerca Biomèdica de Lleida, 25198 Lleida, Spain; ^4^Dialysis Unit, IIS-Fundación Jiménez Díaz, School of Medicine, UAM, Madrid, Spain; ^5^Institute of Renal Research Queen Sophia (IRSIN), Spain; ^6^Division of Nephrology and Hypertension, IIS-Fundación Jiménez Díaz, UAM, Madrid, Spain; ^7^Spanish Biomedical Research Centre in Diabetes and Associated Metabolic Disorders (CIBERDEM), Spain

## Abstract

Chronic kidney disease is characterized by Vitamin D deficiency and activation of the renin-angiotensin-aldosterone system. Increasing data show that vitamin D receptor agonists (VDRAs) exert beneficial effects in renal disease and possess anti-inflammatory properties, but the underlying mechanism remains unknown. Emerging evidence suggests that “a disintegrin and metalloproteinase” (ADAM)/epidermal growth factor receptor (EGFR) signalling axis contributes to renal damage. Aldosterone induces EGFR transactivation regulating several processes including cell proliferation and fibrosis. However, data on tubular epithelial cells is scarce. We have found that, in cultured tubular epithelial cells, aldosterone induced EGFR transactivation via TGF-*α*/ADAM17. Blockade of the TGF-*α*/ADAM17/EGFR pathway inhibited aldosterone-induced proinflammatory gene upregulation. Moreover, among the potential downstream mechanisms, we found that TGF-*α*/ADAM17/EGFR inhibition blocked ERK and STAT-1 activation in response to aldosterone. Next, we investigated the involvement of TGF-*α*/ADAM17/EGFR axis in VDRA anti-inflammatory effects. Preincubation with the VDRA paricalcitol inhibited aldosterone-induced EGFR transactivation, TGF-*α*/ADAM-17 gene upregulation, and downstream mechanisms, including proinflammatory factors overexpression. In conclusion, our data suggest that the anti-inflammatory actions of paricalcitol in tubular cells could depend on the inhibition of TGF-*α*/ADAM17/EGFR pathway in response to aldosterone, showing an important mechanism of VDRAs action.

## 1. Introduction

One of the earliest pathologic features of chronic kidney disease (CKD) patients is active vitamin D deficiency [1, 2]. Increasing data show that vitamin D receptor agonists (VDRAs) therapy decreases proteinuria, may reduce renal damage progression, and improves cardiovascular outcomes in CKD patients [1–3]. These beneficial effects are independent of serum parathyroid hormone, phosphorus, and calcium levels suggesting that vitamin D presents pleotropic actions, beyond mineral metabolism regulation [1, 4]. Active vitamin D (1,25–dihydroxy vitamin D(3) or calcitriol) mediates its biological effects by binding to the vitamin D receptor (VDR), which then translocates to the nuclei of target cells [1]. In experimental renal disease vitamin D or VDRAs treatment diminished fibrosis, mesangial proliferation, podocyte loss, and inflammatory cell infiltration [5–10]. However, the molecular mechanism involved in the anti-inflammatory effects of vitamin D in the setting of CKD remains poorly characterized.

The renin-angiotensin-aldosterone system (RAAS) is a major mediator of progressive renal injury in CKD, with angiotensin II (AngII) and aldosterone (Aldo) being the most relevant RAAS components [11, 12]. Both factors promote renal inflammation, fibrosis, and podocyte injury [13–15]. There is a close relation between vitamin D and the RAAS. The hormonal form of vitamin D is a negative endocrine regulator of the RAAS by suppressing renin biosynthesis [16]. Homozygous VDR knockout mice develop high renin hypertension, cardiac hypertrophy, and increased susceptible to kidney damage following unilateral ureteral obstruction [17, 18]. Therefore, investigation of underlying mechanisms implicated in the relation between RAAS and vitamin D actions in CKD is an important field of research.

Emerging evidence suggests that blockade of epidermal growth factor receptor (EGFR) can be a therapeutic option for renal diseases. Experimental studies have shown that genetic or pharmacological EGFR blockade ameliorates renal disease progression, mainly by diminishing kidney fibrosis [19, 20]. Regarding the RAAS, both AngII and Aldo, after binding to their specific receptors, can transactivate EGFR, via “a disintegrin and metalloproteases” (ADAMs), thus regulating cellular functions, including proliferation, hypertrophy, and migration [21–23]. ADAMs are membrane-spanning metalloproteases involved in cleavage of extracellular substrates (shedding), including EGF family ligands, both constitutively and in response to regulatory stimulation [24, 25]. In the kidney, ADAM17, also known as TACE, participates in the shedding of the EGFR ligands, heparin binding EGF-like growth factor (HB-EGF), and transforming growth factor-*α* (TGF-*α*) [26–29]. Both ligands are involved in AngII-induced EGFR transactivation, although some differences have been described between cell types and tissues [30]. In mice, ADAM17-mediated TGF-*α* shedding contributes to AngII-induced experimental renal fibrosis [20]. Most of the studies on Aldo/EGFR pathway have been done in cultured cells, mainly in vascular smooth muscle cells [14] and in mesangial cells, the latter showing a role in cell proliferation [31]. We now demonstrated here that, in cultured tubular epithelial cells, Aldo activates the EGFR pathway via ADAM-17/TGF-*α* shedding, leading to upregulation of proinflammatory factors. These data are in line with our recent observation that blockade of the ADAM17/EGFR axis prevents experimental renal inflammation induced by systemic administration of the TWEAK cytokine [32], showing that this pathway, besides regulating proliferation and fibrosis, could contribute to renal inflammation. Furthermore, we now show that the beneficial anti-inflammatory effects of VDRAs such as paricalcitol in renal disease may be explained by inhibition of Aldo-mediated proinflammatory factors overexpression through modulation of ADAM17/TGF-*α*/EGFR signalling axis and dampening of downstream mechanisms, including ERK and STAT-1 activation. Our data add novel information about mechanisms involved in the well-known anti-inflammatory properties of VDRAs and contribute to better design of future clinical trials.

## 2. Material and Methods

### 2.1. Cultured Cells

Human kidney proximal tubule epithelial cells (HK2 cell line, ATCC CRL-2190) were grown in RPMI 1640 with 10% fetal bovine serum (FBS), 1% glutamine, 100 U/mL penicillin, 100 *μ*g/mL streptomycin, 5 *μ*g/mL insulin-transferrin-selenite, and 36 ng/mL hydrocortisone in 5% CO_2_ at 37°C. When cells reached 60 to 70% confluence, they were incubated in serum-free medium for 24 hours before the experiments.

Tubuloepithelial proximal murine cells (MCT cell line) were originally obtained from Dr. Eric Neilson (Vanderbilt University) and used for gene expression studies. These cells were grown in RPMI 1640 with 10% FBS, 1% glutamine, 100 U/mL penicillin, and 100 *μ*g/mL streptomycin in 5% CO_2_ at 37°C. When reached 60 to 70% confluence, they were maintained in RPMI with 1% FBS for 24 hours.

Cells were treated with recombinant Aldo (1 *μ*mol/L; Sigma). In some experiments cells were preincubated for 1 hour with the following inhibitors prior to stimulation: the EGFR kinase inhibitor AG1478 (100 nmol/L), the ERK inhibitor U0126 (10 *μ*mol/L; Calbiochem), TAPI-2, a specific inhibitor of ADAM-17 (50 *μ*mol/L, Enzo Life Sciences), and a specific inhibitor of HB-EGF, CRM197 (10 *μ*g/mL, Sigma). In some experiments, cells were preincubated for 24 hours with a neutralizing antibody anti-TGF-*α* (2.5 *μ*g/mL, Abcam) and for 48 hours with paricalcitol (12 *μ*mol/L, Abbott). DMSO was used as a solvent in many of these reagents but had no effect on cell viability or on gene expression levels (data not shown).

### 2.2. Protein Studies

The EGFR phosphorylation status was analysed by Western blotting. Briefly, proteins were obtained using lysis buffer [50 mmol/L Tris-HCl and 150 mol/L NaCl; 2 mmol/L EDTA, 2 mmol/L EGTA, and 0.2% Triton X-100, 0.3% IGEPAL; 10 *μ*L/mL protease inhibitor cocktail; 10 *μ*L/mL PMSF and 10 *μ*L/mL orthovanadate]. Protein content was quantified by the BCA method, using bovine serum albumin (BSA) as standard. Cell lysates (25 *μ*g/lane) were separated on 6% to 12% SDS-polyacrylamide gels under reducing conditions. Samples were transferred to nitrocellulose membranes (Bio-Rad), blocked in 50 mmol/L Tris-HCl, pH 7.5, 150 mmol/L NaCl, 0.05% Tween-20, and 5% milk, and incubated overnight at 4°C with the following antibodies [dilution]: anti-phosphorylated-EGFR on Tyrosine (Y) 1068 (p-EGFR_1068_) [1 : 250] (Calbiochem), ADAM17 [1 : 1000] (Abcam), EGFR [1 : 250], p-ERK1/2 [1 : 200] (Santa Cruz Biotechnology), and p-STAT1 [1 : 500] (Invitrogen). Subsequently, membranes were incubated with a peroxidase-conjugated secondary antibody and developed using the ECL chemiluminescence kit (Amersham Pharmacia Biotech). Protein quality and transfer efficiency were assessed by Ponceau red staining (not shown). To assess protein loading, membranes were incubated with anti-GAPDH [1 : 10000] (Chemicon International). Total proteins were used as control for phosphorylation studies. Films were scanned using the Gel Doc EZ machine imager and analysed using the Image Lab 3.0 (Bio-Rad).

### 2.3. Gene Silencing

The ADAM-17 gene was silenced using an interference silencer siRNA or its corresponding scramble control (Ambion). Subconfluent cells were transfected for 24 hours with 25 nmol/L siRNA using 50 nmol/L of Lipofectamine RNAiMAX (Invitrogen), according to the manufacturer's instructions. Subsequently, cells were incubated with 10% FBS heat inactivated for 24 hours. Then, cells were incubated in serum-free medium for 24 hours before the experiment. The specificity and efficiency of silencing were checked by Western blot with an anti-ADAM-17 antibody (AbCam) [1 : 1000].

### 2.4. Gene Expression Studies

Total RNA was isolated in Trizol (Invitrogen, Groningen, Netherlands) from samples and mouse kidney cells. cDNA was synthesized using the High Capacity cDNA Archive Kit (Applied Biosystems, Foster City, CA) using 2 *μ*g of total RNA with random hexamer primers, following the manufacturer's instructions. We performed real-time PCR expression using probes (Taqman probes labelled with FAM fluorophore) from Applied Biosystems: VDR Mm_00437297_m1; ADAM-17 Mm_00456428_m1; CCL-2 Mm00441242_m1; CCL-5 Mm_01302428_m1; TGF-*α* Mm_00446232_m1. Data were normalized to 18S ribosomal RNA of eukaryotic: 4210893E (VIC). The number of copies of mRNA for each sample was calculated by the instrument software using the Ct. value (shaped point arithmetic analysis on the thermocycler). The results were expressed in relative copy number calculated relative to unstimulated cells or control mice, after normalization to 18S.

### 2.5. Statistical Analysis

The results shown in the text are expressed as mean ± SEM. The differences between the groups treated with agonists and controls were evaluated by the Student's *t*-test and Mann-Whitney test, and *P* < 0.05 was considered significant. Statistical analysis was performed using SPSS statistical software (version 11.0, Chicago, IL).

## 3. Results

### 3.1. Aldosterone Induces EGFR Transactivation via ADAM17 and Subsequent Release of TGF-*α* in Cultured Tubular Epithelial Cells

**Figure 1:**
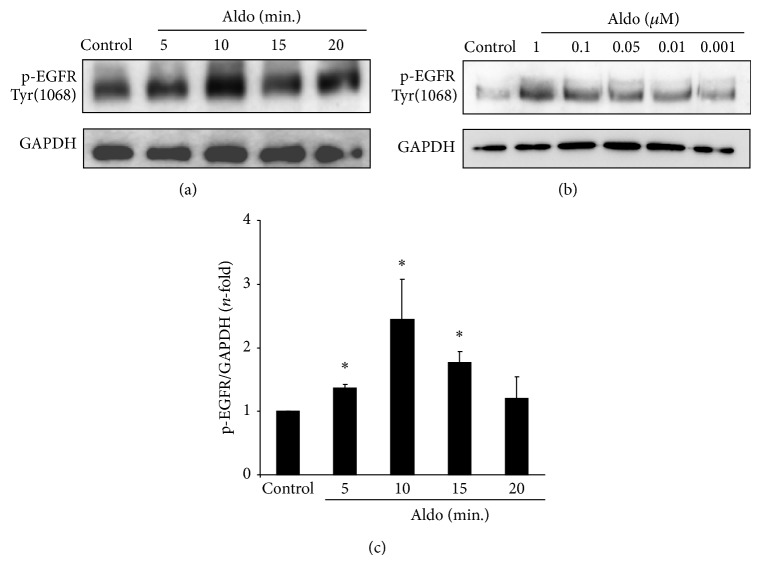
Aldosterone induces EGFR phosphorylation in cultured tubular epithelial cells. Human tubular epithelial cells were treated (a) with 1 *μ*mol/L aldosterone (Aldo) for increasing times or (b) with several concentrations (range 1–0.001 *μ*mol/L Aldo) for 15 min. (c) The figure shows a quantification of time-course EGRF phosphorylation. The data are expressed as mean ± SEM of 4 independent experiments. ^*^ *P* < 0.05 versus control.

**Figure 2:**
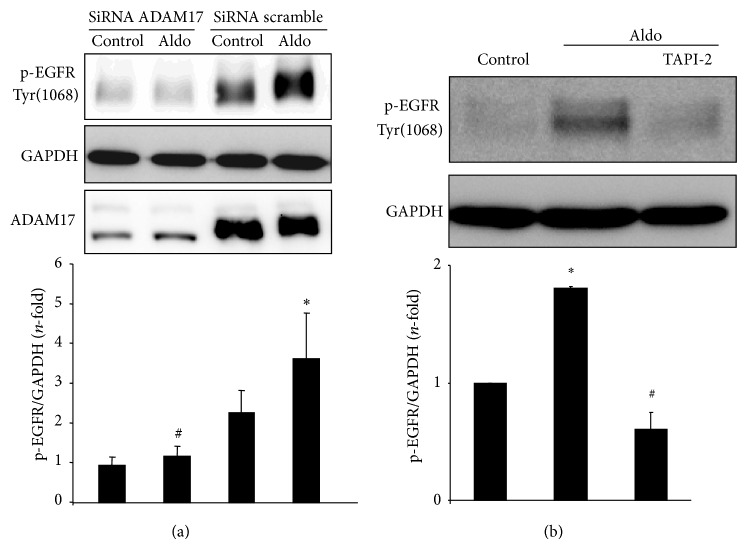
ADAM17 inhibition blocks aldosterone-mediated EGFR activation in tubular epithelial cells. HK2 cells were transfected with an ADAM17 siRNA or scrambled siRNA, as described in “methods,” before stimulation with 1 *μ*mol/L Aldo for 15 min. Phosphorylated-EGFR levels were evaluated by Western blot using an antibody against Y1068 phosphorylated-EGFR (p-EGFR1068). GAPDH levels were used as loading control and ADAM17 as silencing control. (a) Representative Western blot and quantification of data expressed as mean ± SEM of 4 independent experiments. ^*^ *P* < 0.05 versus untreated siRNA control-transfected cells control. ^#^ *P* < 0.05 versus Aldo-treated siRNA scramble cells. (b) Cells were preincubated with the specific ADAM17 inhibitor TAPI-2 (50 *μ*mol/L) for 1 hour and then stimulated with 1 *μ*mol/L Aldo for 15 min. Representative Western blot and data expressed as mean ± SEM of 4 independent experiments. ^*^ *P* < 0.05 versus control. ^#^ *P* < 0.05 versus Aldo.

Aldo induced EGFR transactivation in several cells, including epithelial cells and mesangial cells [31, 33]. In cultured human tubular epithelial cells (HK2 cell line), Aldo induced a rapid activation of EGFR, as shown by increased EGFR phosphorylation on Tyr1068, in a time and dose-dependent manner, presenting a maximal effect at 1 *μ*mol/L Aldo after 10 min (Figures 1(a), 1(b) and 1(c)). The blockade of ADAM17, by gene silencing or pharmacological inhibition using TAPI-2, markedly diminished Aldo-induced EGFR phosphorylation (Figures 2(a) and 2(b)).

**Figure 3:**
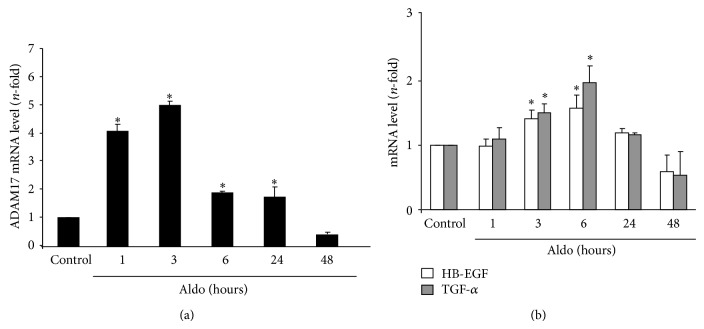
Aldosterone regulates genes expression of ADAM17 (a), TGF-*α*, and HB-EGF (b). Cells were treated with 1 *μ*mol/L Aldo for increasing times and gene expression levels were evaluated by real-time PCR. Data are expressed as mean ± SEM of 3 independent experiments. ^*^ *P* < 0.05 versus control.

ADAM17 mRNA is constitutively expressed in normal adult human kidneys and is increased in disease conditions [34]. In cultured tubular epithelial cells stimulation with Aldo rapidly increased ADAM17 gene expression, which remained elevated up to 24 hours (Figure 3(a)). Among the EGFR ligands, HB-EGF and TGF-*α* are released by ADAM17 and have a role in renal diseases. In tubular epithelial cells, Aldo upregulated gene expression of HB-EGF and TGF-*α*, observed after 3 hours (Figure 3(b)).

**Figure 4:**
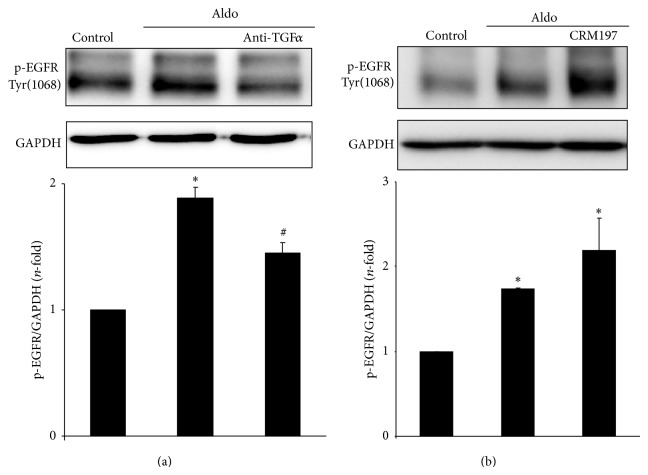
Aldosterone transactivates EGFR via TGF*α*, but not HB-EGF, shedding. Cells were pretreated for 24 h with a neutralizing antibody against TGF*α* (2.5 *μ*g/mL) (a) or 1 hour with the HB-EGF pharmacological inhibitor CRM197 (10 *μ*mol/L) (b) before stimulation with 1 *μ*mol/L Aldo for 15 min. Western blot experiment and data are expressed as mean ± SEM of 3 or 5 independent experiments, respectively. ^*^ *P* < 0.05 versus control. ^#^ *P* < 0.05 versus Aldo.

TGF-*α* blockade, using a specific neutralizing antibody, inhibited EGFR phosphorylation in response to Aldo in tubular cells (Figure 4(a)). In contrast, the pharmacological inhibition of HB-EGF by CRM197, a nontoxic mutant of diphtheria toxin that neutralizes HB-EGF binding to EGFR [29], had no effect on Aldo-mediated EGFR activation (Figure 4(b)).

### 3.2. Aldosterone Regulates Proinflammatory Gene Expression via the ADAM-17/TGF-*α*/EGFR Axis in Cultured Tubular Epithelial Cells

**Figure 5:**
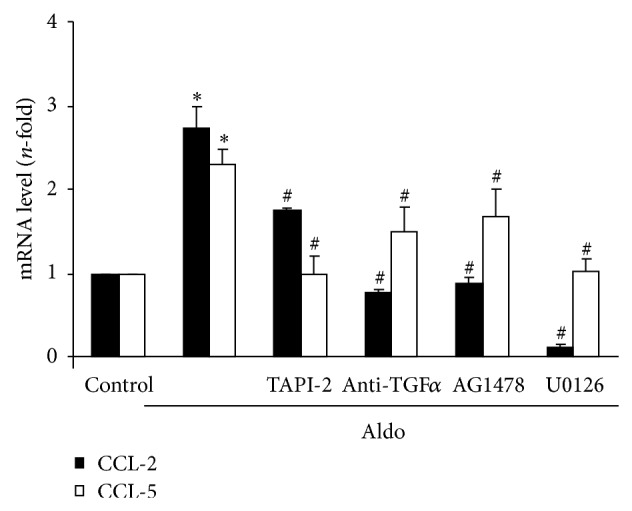
ADAM17/TGF-*α*/EGFR signaling blockade inhibits aldosterone-mediated proinflammatory factors upregulation. Cells were pretreated with the specific ADAM17 inhibitor TAPI-2 (50 *μ*mol/L; 1 hour), a neutralizing antibody against TGF*α* (2.5 *μ*g/mL, 24 hour), the EGFR kinase inhibitor AG 1478 (100 nmol/L, 1 hour), or the ERK inhibitor U0126 (10 *μ*mol/L; 1 hour) before stimulation with 1 *μ*mol/L Aldo for 6 hours. CCL-2 and CCL-5 gene expression levels were determined by real-time PCR. Data are expressed as mean ± SEM of 2-3 experiments. ^*^ *P* < 0.05 versus control. ^#^ *P* < 0.05 versus Aldo.

Aldo exerts proinflammatory actions in the kidney, including the regulation of proinflammatory gene expression in cultured cells [35]; however, the role of the ADAM-17/EGFR pathway in these Aldo-mediated responses has not been evaluated. In cultured tubular epithelial cells, we have blocked ADAM-17/TGF-*α*/EGFR pathway using the following inhibitors: the ADAM17 inhibitor TAPI-2, an anti-TGF-*α* neutralizing antibody, and the EGFR kinase inhibitor AG1478. All of these inhibitors significantly diminished Aldo-induced gene upregulation of the proinflammatory factors CCL-2 and CCL-5 (Figure 5).

### 3.3. Aldosterone Activates Several Intracellular Mechanisms via the ADAM-17/TGF-*α*/EGFR Axis

**Figure 6:**
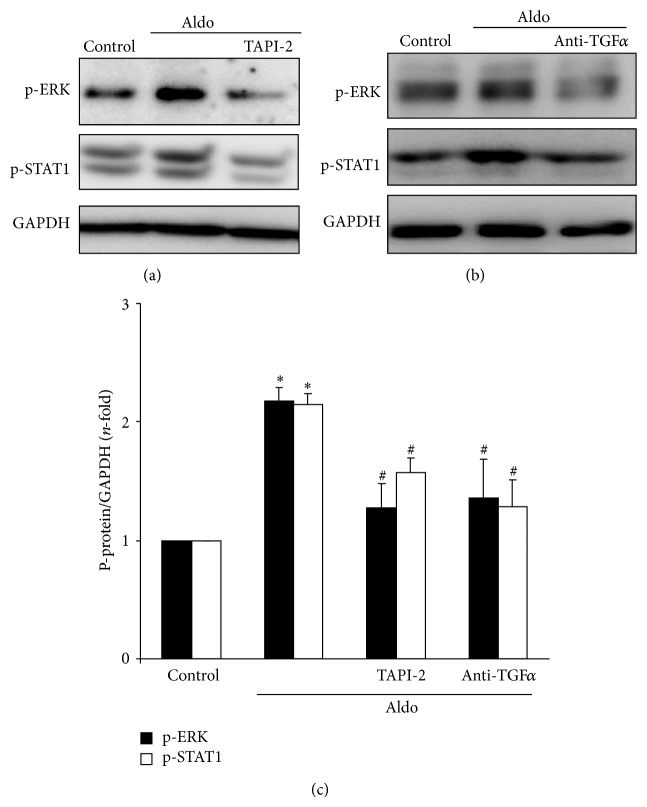
Aldosterone activates ERK and STAT pathways via ADAM-17/TGF-*α*/EGFR axis. HK2 cells were preincubated with (a) the specific ADAM17 inhibitor TAPI-2 (50 *μ*mol/L; 1 hour) or (b) a neutralizing antibody against TGF*α* (2.5 *μ*g/mL, 24 hour) and then stimulated with 1 *μ*mol/L Aldo for 15 min. Figures (a) and (b) show representative Western blot experiment and (c) data expressed as mean ± SEM of 3 experiments. ^*^ *P* < 0.05 versus control. ^#^ *P* < 0.05 versus Aldo.

Among the EGFR downstream signalling mechanisms, activation of the MAPK cascade has special relevance in the regulation of inflammatory events. Aldo increased EGFR phosphorylation on tyrosine 1068, which has been previously involved in ERK signalling [29, 34]. In human tubular cells, Aldo increased ERK phosphorylation and ERK blockade diminished Aldo-induced proinflammatory gene overexpression (Figure 5). Moreover, ERK activation was prevented when the ADAM-17/TGF-*α*/EGFR pathway was inhibited using the above-described specific blockers (Figures 6(a), 6(b) and 6(c)). Aldo also activated STAT-1 via the ADAM-17/TGF-*α*/EGFR pathway (Figures 6(a), 6(b) and 6(c)).

### 3.4. Paricalcitol Inhibits Aldosterone-Induced Proinflammatory Gene Expression in Cultured Renal Cells by Modulating the ADAM-17/TGF-*α*/EGFR Axis

**Figure 7:**
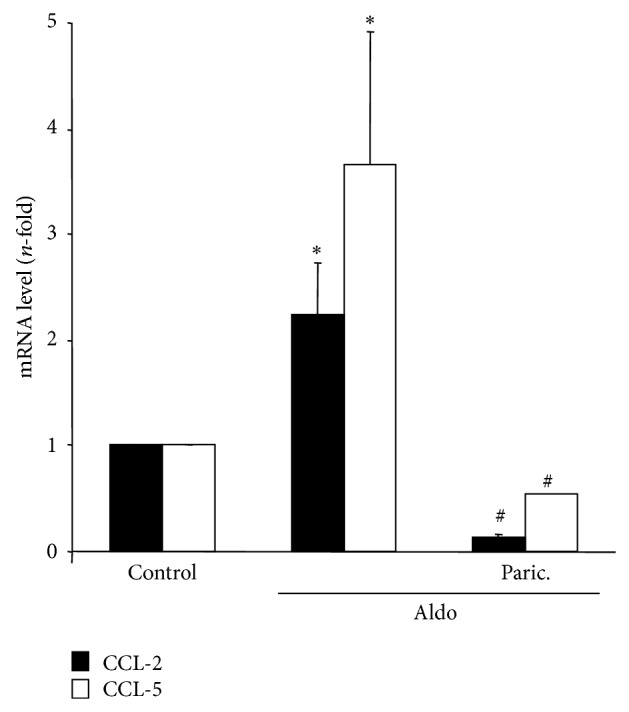
Paricalcitol inhibits aldosterone-induced proinflammatory genes in cultured tubular epithelial cells. Cells were pretreated with the analogue of vitamin D paricalcitol (12 *μ*mol/L) for 48 hours before stimulation with 1 *μ*mol/L Aldo for 6 hours. Gene expression levels were determined by real-time PCR. Data are expressed as mean ± SEM of 3 independent experiments. ^*^ *P* < 0.05 versus control. ^#^ *P* < 0.05 versus Aldo.

In tubular epithelial cells, preincubation with the VDRA paricalcitol for 48 hours, inhibited proinflammatory genes induction caused by Aldo (Figure 7). These data confirm the anti-inflammatory properties of paricalcitol.

**Figure 8:**
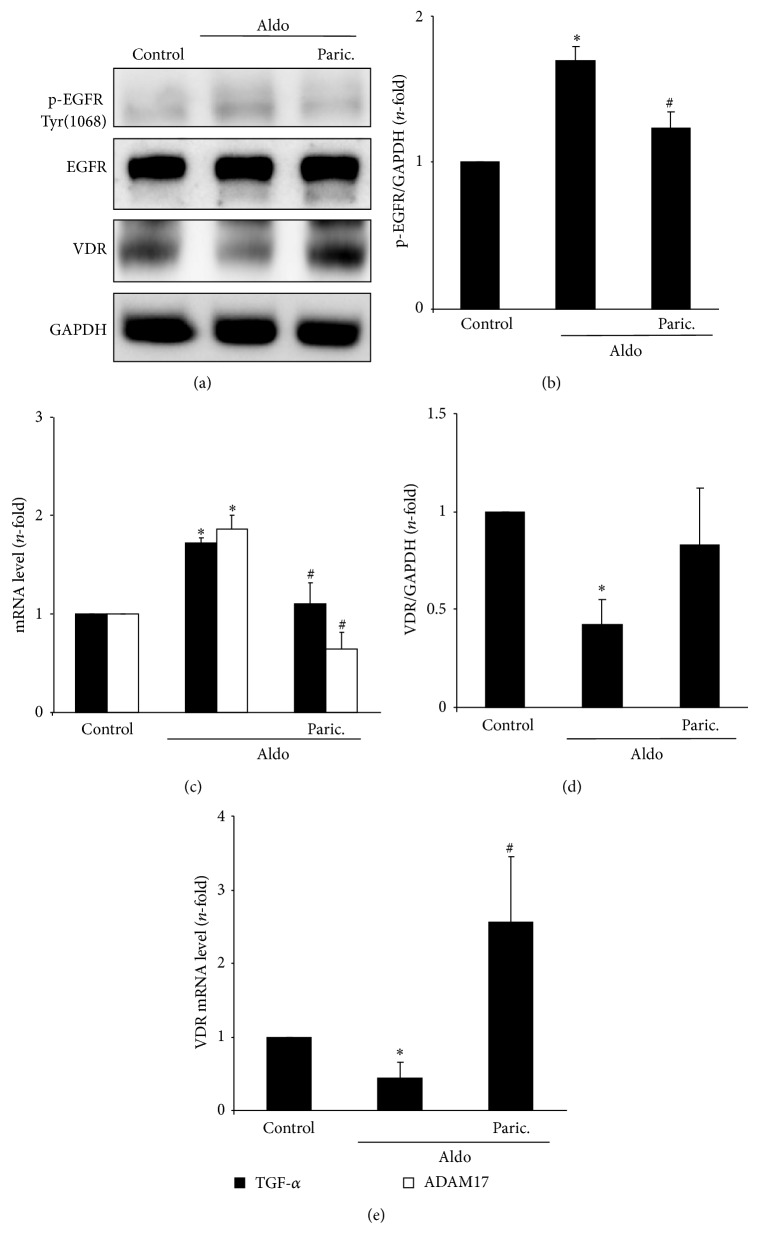
Paricalcitol inhibits aldosterone-induced activation of ADAM-17/TGF-*α*/EGFR signalling pathway. Cells were pretreated with the analogue of vitamin D Paricalcitol (12 *μ*mol/L) for 48 hours before stimulation with 1 *μ*mol/L Aldo for 15 min (protein) or 6 hours (gene studies). Paricalcitol inhibits Aldo-induced EGFR activation (a, b) and gene overexpression of TGF*α* and HB-EGF (c). Figure (a) shows a representative Western blot, in (b) quantification of p-EGFR and in (c) gene expression levels by real-time PCR. Paricalcitol restores Aldo-induced changes in VDR protein (a, d) and gene (e) expression levels. Figure (a) shows a representative Western blot, in (d) quantification of VDR protein levels and in (e) gene expression levels. Data are expressed as mean ± SEM of 3 independent experiments. ^*^ *P* < 0.05 versus control. ^#^ *P* < 0.05 versus Aldo.

Paricalcitol blocked Aldo-induced EGFR transactivation, as shown by the downregulation of phosphorylated-EGFR levels (Figures 8(a) and 8(b)) and ADAM-17/TGF-*α* gene overexpression (Figure 8(c)) to control values.

During renal damage, VDR expression is downregulated [1, 3]. Interestingly, in tubular epithelial cells Aldo downregulated VDR gene and protein levels (Figures 8(a), 8(d), and 8(e)). Pretreatment with paricalcitol restored VDR levels, indicating that beneficial effects of paricalcitol could be due to modulation of VDR.

**Figure 9:**
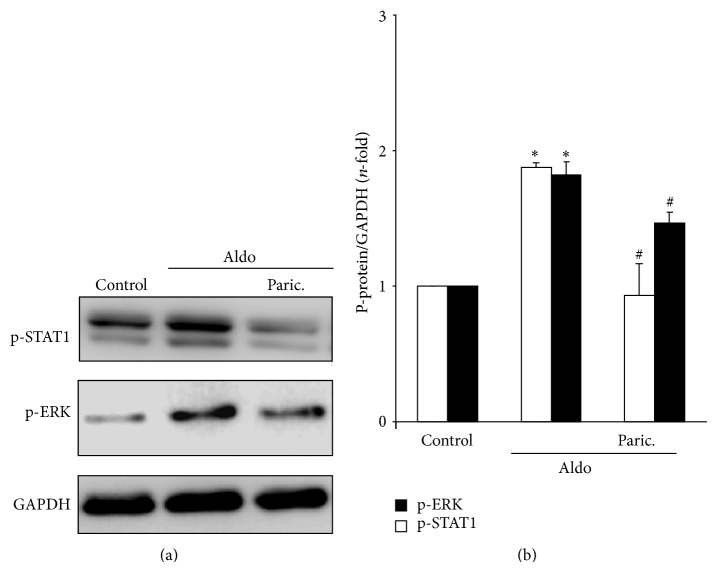
Paricalcitol inhibits aldosterone-mediated activation of ERK and STAT-1 pathway. HK2 cells were preincubated with paricalcitol (12 *μ*mol/L) for 48 hours before stimulation with 1 *μ*mol/L Aldo for 15 min. Figure (a) shows a representative experiment and in (b) quantification of p-ERK and p-STAT1 expressed as mean ± SEM of 3 independent experiments. ^*^ *P* < 0.05 versus control. ^#^ *P* < 0.05 versus Aldo.

Finally, paricalcitol also blocked ERK and STAT-1 activation in response to Aldo stimulation (Figure 9).

## 4. Discussion

**Figure 10:**
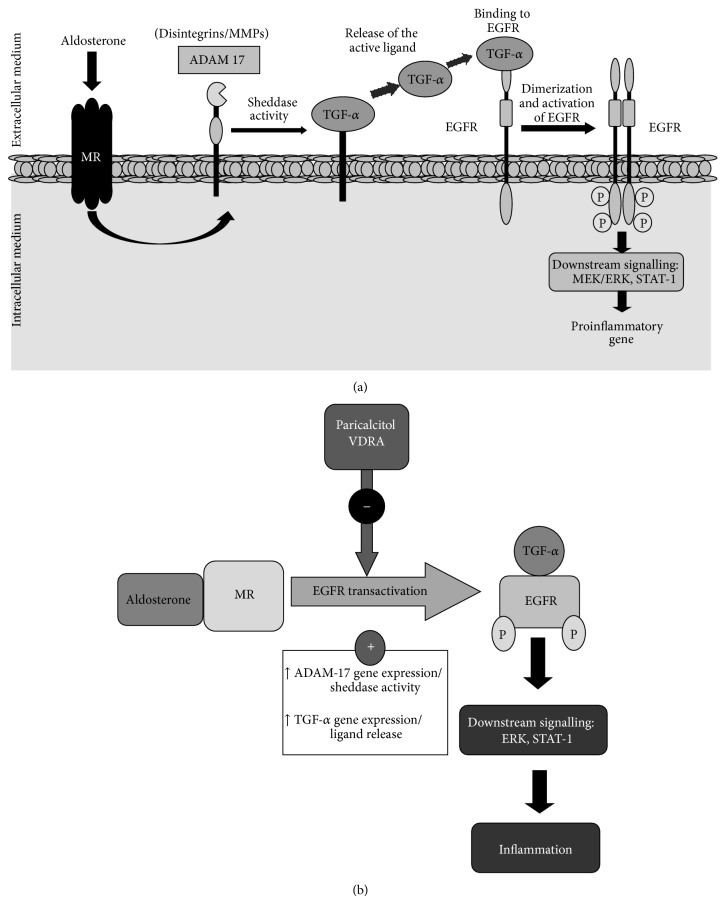
Aldosterone-induced inflammatory responses: role of the ADAM-17/TGF-*α*/EGFR axis and modulation by paricalcitol. (a) Aldosterone activates the ADAM-17/TGF-*α*/EGFR axis to regulate proinflammatory factors. (b) Paricalcitol inhibits aldosterone-induced activation of ADAM-17/TGF-*α*/EGFR signalling pathway. MR: mineralocorticoid receptor; VDRA: vitamin D receptor activators.

Chronic inflammation is a main feature of CKD. Among the factors involved in the inflammatory response in the kidney, local activation of RAAS has special relevance. AngII, the main effector peptide of this system, has been extensively demonstrated to promote renal inflammation [11]. There is previous evidence that Aldo also contributes to this process. Multiple experimental studies in models of hypertension, renal damage, and heart failure have demonstrated that selective Aldo blockade by eplerenone attenuates tissue injury in part by reducing inflammation in Aldo target organs [13, 15, 35]. Treatment with an aldosterone synthase inhibitor ameliorated experimental diabetic nephropathy by decreasing renal inflammation, matrix formation, and albuminuria [36]. Data presented here demonstrate that the ADAM-17/TGF-*α*/EGFR axis is an important mechanism involved in the regulation of proinflammatory factors by Aldo in cultured tubular epithelial cells (Figure 10(a)). Other data also support the involvement of the EGFR/ADAM17 axis in inflammation [37, 38], as we have recently described in TWEAK-mediated experimental renal inflammation [32]. In addition, targeting ADAM17 by pharmacologic inhibition or gene knockout attenuates the inflammatory response in animal models of vascular damage, including hypertension, atherosclerosis, and pulmonary vascular inflammation [21, 38–40].

EGFR transactivation is regulated by ligand sheddase activity [41]. Thus far, 12 EGFR ligands have been described, among them TGF-*α*, HB-EGF, amphiregulin, and connective tissue growth factor could be relevant in renal pathology [41–43]. Regarding the kidney, EGF modulates glomerular hemodynamics and renal metabolism, while TGF-*α*, HB-EGF, and amphiregulin participate in cell survival/proliferation [41, 43]. Moreover, TGF-*α* has been involved in genetic susceptibility to renal disease [44] and in AngII-mediated experimental renal fibrosis [20]. HB-EGF contributes to cell regeneration and repair after ischemia [41]. In HB-EGF-deficient mice with progressive glomerulonephritis, inflammatory renal infiltration and albuminuria were lower which was ascribed to EGFR pathway inhibition in podocytes [45]. In cultured tubular cells, we have found that Aldo upregulates both EGFR ligands, TGF-*α* and HB-EGF, but only TGF-*α* mediates Aldo-induced EGFR transactivation, at least at the time point evaluated. This illustrates that EGFR ligands involved in transactivation are cell and stimuli specific and indicates that future studies are needed to evaluate the EGFR ligands involved in Aldo actions in the kidney. In human renal and cardiovascular pathophysiology, ADAM17 expression may be increased [21, 34, 46]. Our findings suggest that Aldo is one of the drivers of increased ADAM17 expression in tubular cells. Moreover, ADAM17 SNPs have been associated to increases in cardiovascular mortality [47].

Activation of the RAAS has been associated to activation of EGFR signalling in renal and cardiovascular diseases. AngII via EGFR pathway regulates hypertrophy and fibrosis in the kidney [20]. In porcine renal proximal tubular cells HB-EGF shedding-dependent EGFR transactivation regulates AngII-induced cell hypertrophy [48]. Interestingly, Smad activation, the main pathway controlling fibrosis, is independent of HB-EG/EGFR pathway [48]. In mesangial cells, Aldo activates EGFR linked to ROS production, ERK signalling, and modulating cell growth [31], as described in myocytes [49] and vascular cells [50]. In other cell types, Aldo/EGFR may signal through PI3-kinase/Akt/mTOR/p70S6 K1 [22]. We have described here that, in tubular cells, Aldo activates ADAM17 leading to rapid and significant activation of EGFR, which in turn activates downstream cascades, such as the MAPK ERK kinase and the STAT-1 transcription factor (Figure 10(a)).

Several lines of evidence have suggested a potential anti-inflammatory activity of vitamin D in CKD [6, 19]. In experimental glomerular diseases and obstructive nephropathy, administration of vitamin D reduces inflammatory cell infiltration [6–9, 20]. Vitamin D and VDRAs may exert their immunomodulatory actions by direct modulation of immune cells, including macrophages, dendritic cells, and T cells [51]. Vitamin D modulates dendritic cells' maturation and function, the population and function of FOXP3+ and IL-10–producing T regulatory cells [52], and regulates Th17 differentiation and decreasses IL-17A production [53]. Besides regulating immune cells, VDRAs could also exert anti-inflammatory actions by modulating responses of resident renal cells. In this sense, we have found that, in tubular epithelial cells, paricalcitol inhibits Aldo-induced proinflammatory genes upregulation. The mechanisms involved in these anti-inflammatory effects of vitamin D in epithelial cells are not well known. In monocytes/macrophages, vitamin D inhibits LPS-induced cytokine production by upregulating MAPK phosphatase-1 that inactivates p38 and JNK [54]. In tubular cells, we found that paricalcitol inhibited Aldo-induced activation of ERK and STAT-1 (Figure 10(b)), identifying a novel mechanisms of action of VDRAs.

Earlier studies showed a relation between vitamin D and EGFR signalling pathways. Those studies were focused on EGFR binding and regulation of its gene expression [55–58]. After that, many studies demonstrated that vitamin D or VDRAs increase cell proliferation via EGFR in different cell types, by a mechanism that includes changes in EGFR membrane trafficking and downregulation of EGFR growth signalling [1, 59]. However, recent studies suggest that this antiproliferative effect could be mediated by the modulation of the TGF-*α*/EGFR autocrine growth loop [60]. Our results show that in cultured tubular epithelial cells, the VDRA agonist paricalcitol inhibited the EGFR pathway activated by Aldo by modulating TGF-*α*/ADAM17/EGFR signalling pathway activation and expression, supporting this hypothesis.

## 5. Conclusions

Our* in vitro* data in tubular cells stimulated with Aldo suggest that the anti-inflammatory properties of the VDRA paricalcitol are, at least in part, mediated by inhibition of the ADAM-17/TGF-*α*/EGFR and downstream signals, including ERK and STAT-1. In renal diseases, local activation of RAAS contributes to inflammation and tissue damage. Thus, our results show a novel signalling pathway that could be involved in the observed anti-inflammatory effects of paricalcitol in CKD and expand the current understanding of the mechanisms involved in the renoprotective effects of vitamin D and its analogs.
